# Supplementary material for: Neural geometry from mixed sensorimotor selectivity for predictive sensorimotor control
Source: eLife. 2025 May 1;13:RP100064. doi: 10.7554/eLife.100064 (PMC12045623; doi:10.7554/eLife.100064)
Supplement: Supplementary file 1. [file elife-100064-supp1.docx]

| **No.** | **Monkey** | **Session** | **Task condition** | **n (Trials)** | **N (Neurons)** | **Hand trajectory** |
| --- | --- | --- | --- | --- | --- | --- |
| 1 | C | 20221022 | 0 °/s, ±120 °/s, ±240 °/s | 772 | 95 | Yes |
| 2 | C | 20221023 | 0 °/s, ±120 °/s, ±180 °/s, ±240 °/s, ±360 °/s | 1257 | 97 | Yes |
| 3 | C | 20221024 | 0 °/s, ±120 °/s, ±180 °/s, ±240 °/s, ±360 °/s | 1301 | 90 | Yes |
| 4 | C | 20221025 | 0 °/s, ±120 °/s, ±180 °/s, ±240 °/s, ±360 °/s | 1378 | 100 | No |
| 5 | C | 20221026 | 0 °/s, ±180 °/s, ±360 °/s | 801 | 86 | No |
| 6 | C | 20221105 | 0 °/s, ±120 °/s, ±240 °/s | 856 | 68 | No |
| 7 | C | 20221117 | 0 °/s, ±120 °/s, ±240 °/s | 802 | 58 | Yes |
| 8 | G | 20190913 | 0 °/s, ±120 °/s, ±240 °/s | 903 | 85 | Yes |
| 9 | G | 20190914 | 0 °/s, ±120 °/s, ±240 °/s | 855 | 96 | Yes |
| 10 | G | 20190915 | 0 °/s, ±120 °/s, ±240 °/s | 801 | 86 | Yes |
| 11 | G | 20190916 | 0 °/s, ±120 °/s, ±240 °/s | 782 | 123 | No |
| 12 | D | 20180606 | 0 °/s, ±120 °/s, ±240 °/s | 752 | 39 | No |
| 13 | D | 20180701 | 0 °/s, ±120 °/s, ±240 °/s | 458 | 39 | No |
| 14 | D | 20180804 | 0 °/s, ±120 °/s, ±240 °/s | 772 | 55 | No |
| 15 | D | 20180805 | 0 °/s, ±120 °/s, ±240 °/s | 587 | 45 | No |
