# Supplementary material for: Neural geometry from mixed sensorimotor selectivity for predictive sensorimotor control
Source: eLife. 2025 May 1;13:RP100064. doi: 10.7554/eLife.100064 (PMC12045623; doi:10.7554/eLife.100064)
Supplement: Supplementary file 2. [file elife-100064-supp2.docx]

| **Pairs** | **CC1** | **CC2** | **CC3** | **Disparity** |
| --- | --- | --- | --- | --- |
| C-M1-MO vs. C-M1-TO | 0.84 | 0.80 | 0.64 | 0.88 |
| C-M1-MO-1 vs. C-M1-MO-2 | 0.95 | 0.90 | 0.85 | 0.63 |
| C-M1-MO vs. C-PMd-MO | 0.90 | 0.75 | 0.73 | 0.83 |
| C-M1-MO vs. G-M1-MO-1 | 0.93 | 0.87 | 0.79 | 0.71 |
| C-M1-MO vs. G-M1-MO-2 | 0.93 | 0.89 | 0.79 | 0.70 |
| G-M1-MO-1 vs. G-M1-MO-2 | 0.97 | 0.87 | 0.82 | 0.51 |
| C-M1-MO vs. RNN-MO | 0.95±0.00 | 0.93±0.00 | 0.79±0.02 | 0.88±0.01 |
| G-M1-MO-1 vs. RNN-MO | 0.97±0.00 | 0.89±0.00 | 0.86±0.01 | 0.82±0.01 |
| G-M1-MO-2 vs. RNN-MO | 0.98±0.00 | 0.91±0.00 | 0.87±0.01 | 0.82±0.01 |
| C-M1-MO vs. RNN-MO-shuffle | 0.15±0.00 | 0.10±0.00 | 0.09±0.00 | 1.00±0.00 |
