# Supplementary material for: Neural geometry from mixed sensorimotor selectivity for predictive sensorimotor control
Source: eLife. 2025 May 1;13:RP100064. doi: 10.7554/eLife.100064 (PMC12045623; doi:10.7554/eLife.100064)
Supplement: Supplementary file 3. [file elife-100064-supp3.docx]

|  | **Distance Error** | **R^2^ of fitting ellipses** |
| --- | --- | --- |
| Intact models | 0.0046 ± 0.0027 | 0.9781 ± 0.0498 |
| **Ablated nodes** |  |  |
| S | 0.1358±0.0147 | 0.9351±0.0856 |
| G | 0.1438±0.0141 | 0.9487±0.0644 |
| A | 0.1533±0.0184 | 0.9510±0.0642 |
| S only | 0.0969±0.0278 | 0.9019±0.0904 |
| G only | 0.0752±0.0346 | 0.9448±0.0707 |
| A only | 0.1133±0.0335 | 0.9337±0.0797 |
| **Changed connections** |  |  |
| S → S | 0.0697 ± 0.0279 | 0.8361 ± 0.0975 |
| S → G | 0.0687 ± 0.0300 | 0.8891 ± 0.0956 |
| S → A | 0.0815 ± 0.0311 | 0.8473 ± 0.1140 |
| G → S | 0.0670 ± 0.0294 | 0.8499 ± 0.1051 |
| G → G | 0.0598 ± 0.0263 | 0.8646 ± 0.1161 |
| G → A | 0.0773 ± 0.0308 | 0.8321 ± 0.1139 |
| A → S | 0.0763 ± 0.0287 | 0.8415 ± 0.1063 |
| A → G | 0.0777 ± 0.0320 | 0.8440 ± 0.1198 |
| A → A | 0.0803 ± 0.0296 | 0.7818 ± 0.1265 |
