# Supplementary material for: Neural geometry from mixed sensorimotor selectivity for predictive sensorimotor control
Source: eLife. 2025 May 1;13:RP100064. doi: 10.7554/eLife.100064 (PMC12045623; doi:10.7554/eLife.100064)
Supplement: Supplementary file 4. [file elife-100064-supp4.docx]

| **Models** | **GM** | **GT** | **sINIT** |
| --- | --- | --- | --- |
| Performance | 0.0052±0.0032 | 0.0130±0.0075 | 0.0131±0.0071 |
| Correction rate | 1.00±0.01 | 0.84±0.13 | 0.82±0.15 |
| Gain (G) nodes % | 51.9±5.7% | 68.0±6.1% | 50.8±12.9% |
| PD shift (S) nodes % | 18.7±6.2% | 56.1±3.3% | 48.7±15.0% |
| Addition (A) nodes % | 68.6±5.8% | 61.9±5.4% | 61.0±8.8% |
| No modulation nodes % | 12.3±5.4% | 7.1±3.5% | 10.7±6.9% |
| No activation nodes % | 40.6±3.9% | 62.1±31.0% | 70.7±24.0% |
| PC1 explained variance % | 54.2±3.0% | 38.1±2.3% | 52.6±6.9% |
| PC2 explained variance % | 42.4±2.3% | 28.1±2.8% | 32.1±5.9% |
| PC3 explained variance % | 2.4±1.0% | 14.3±2.6% | 7.1±1.5% |
| R^2^ of fitting ellipses | 0.9664±0.0281 | 0.7616±0.1655 | 0.8507±0.0687 |
| R^2^ of fitting ellipses (Dim 1) | 0.9909±0.0173 | 0.8365±0.1365 | 0.8905±0.0512 |
| R^2^ of fitting ellipses (Dim 2) | 0.9880±0.0214 | 0.7857±0.2105 | 0.8781±0.0683 |
| R^2^ of fitting ellipses (Dim 3) | 0.0170±0.0151 | 0.4314±0.3281 | 0.3722±0.3400 |
| R^2^ of fitting tilting angle | 0.0062 | 0.2528 | 0.4614 |
